# Correction: Mathematical modeling suggests heterogeneous replication of *Mycobacterium tuberculosis* in rabbits

**DOI:** 10.1371/journal.pcbi.1013048

**Published:** 2025-04-25

**Authors:** Vitaly V. Ganusov, Afsal Kolloli, Selvakumar Subbian

In the Funding statement, the grant number of the author Selvakumar Subbian (SS) is listed incorrectly. The correct grant number is: R01AI161822.
