# Supplementary figures and images for: Mathematical modeling suggests heterogeneous replication of Mycobacterium tuberculosis in rabbits
Source: PLoS Comput Biol. 2024 Nov 25;20(11):e1012563. doi: 10.1371/journal.pcbi.1012563 (PMC11627432; doi:10.1371/journal.pcbi.1012563)

**A**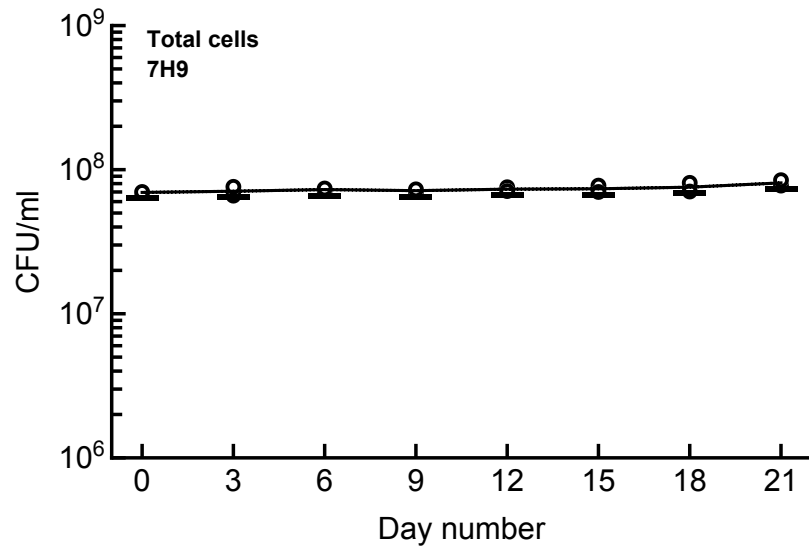**B**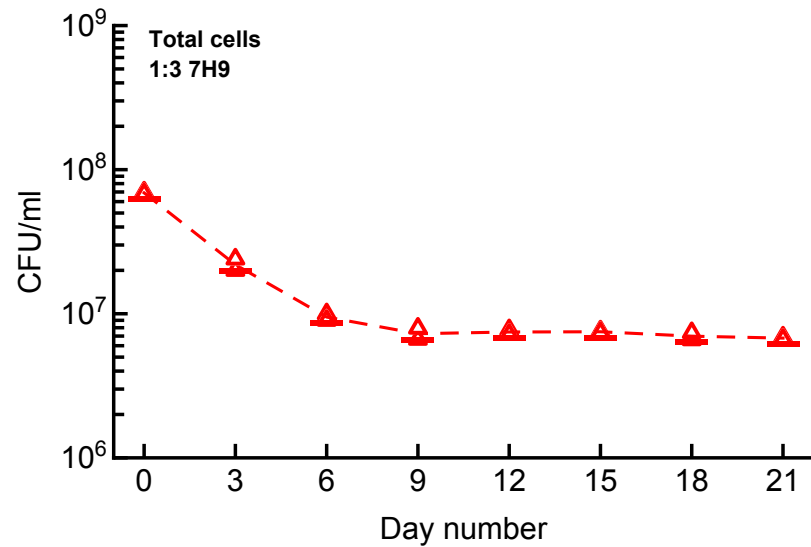**C**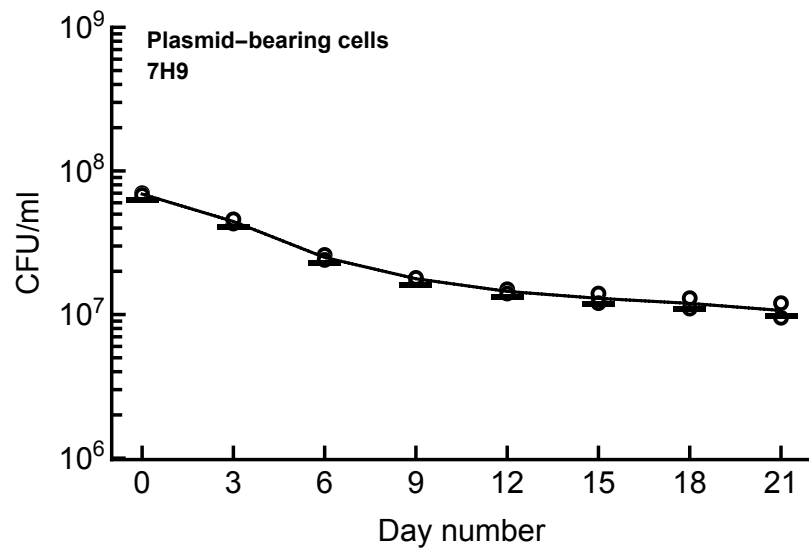**D**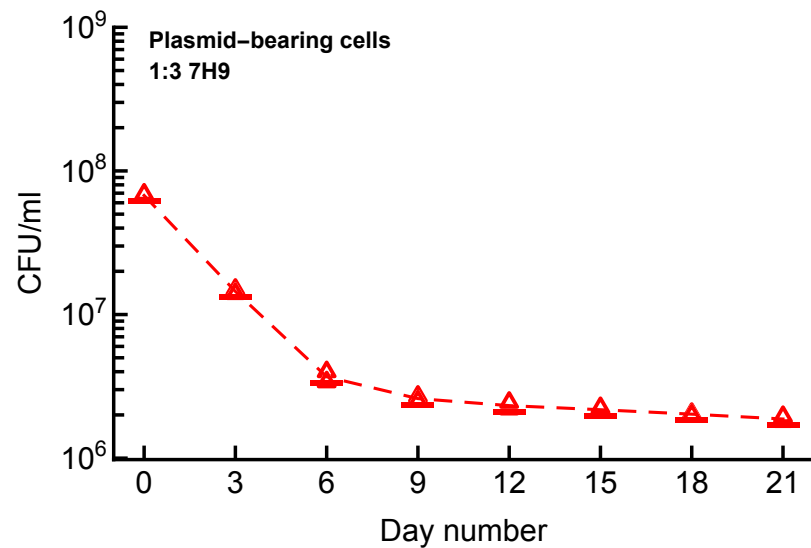**E**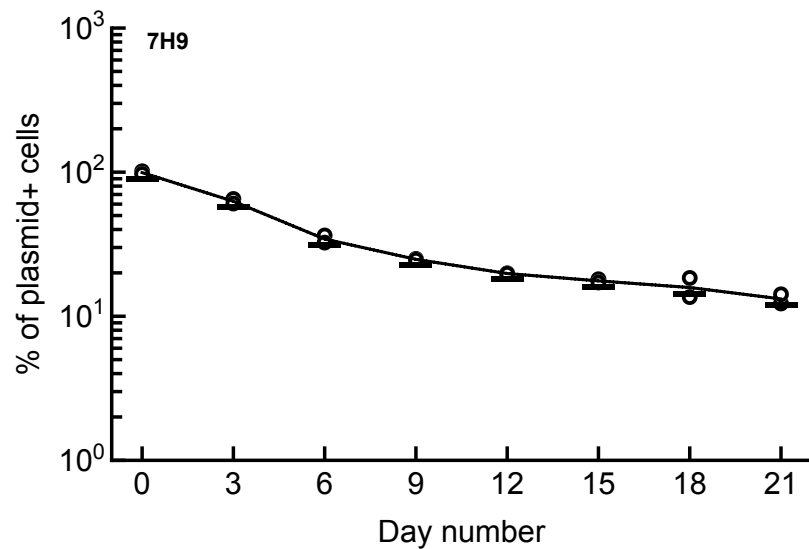**F**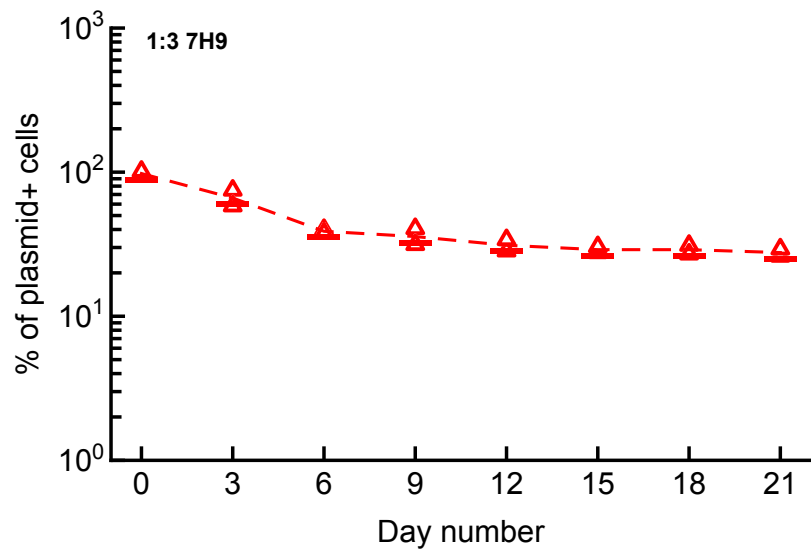

Supplement: S1 Fig — We performed new experiments in which Mtb strain HN878-pBP10, carrying an unstable plasmid pBP10, was cultured in complete 7H9 (panels A, C, E) or 1:3 diluted 7H9 (panels B, D, F) media for 3 days and transferred to new media. We determined concentration of the total number of bacteria (A-B), of the number of plasmid bearing cells per mL (C-D), or the percent of plasmid-bearing cells in the population (E-F) at the end of each 3 day culture. (PDF) [file pcbi.1012563.s001.pdf]

**A**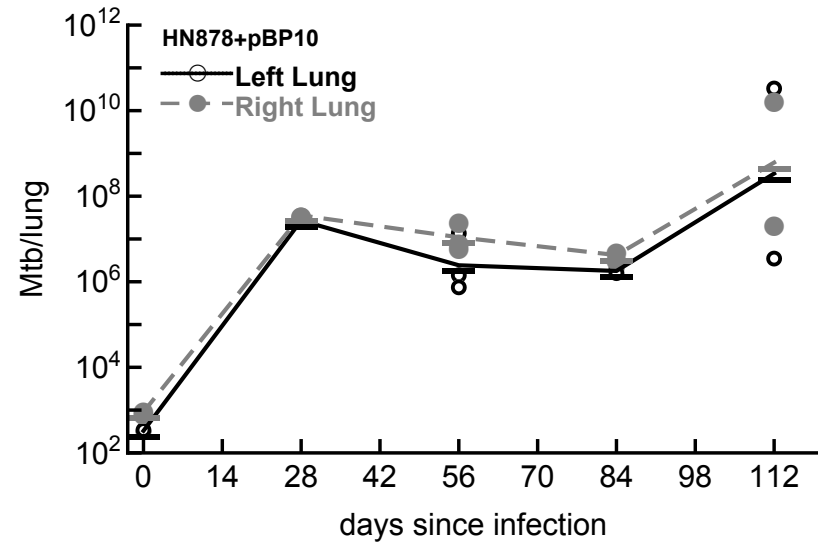**B**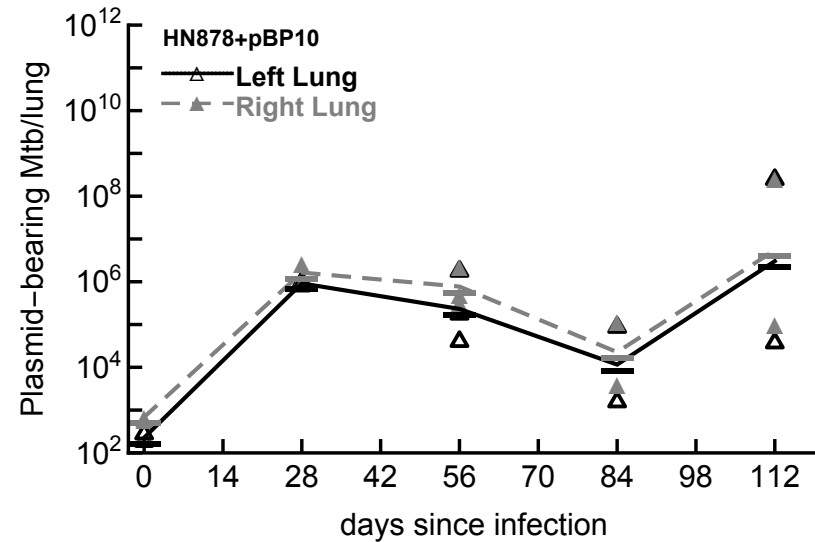**C**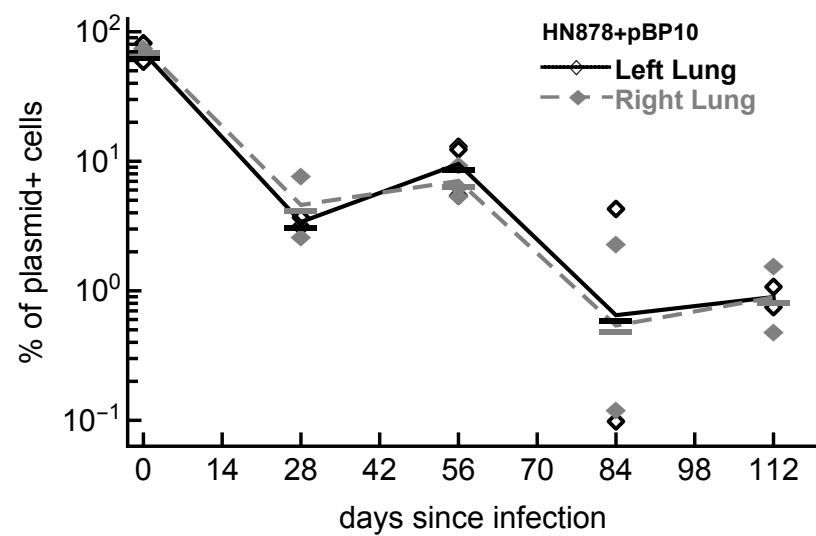

Supplement: S2 Fig — We plot the data are for the total number of bacteria and number of plasmid-bearing bacteria in the lungs (panels A and B) or the percent of plasmid-bearing cells in the population (panel C) separately for the left and right lung. Markers denote measurements for individual rabbit lungs and lines connect geometric means of bacterial counts. (PDF) [file pcbi.1012563.s002.pdf]

**A**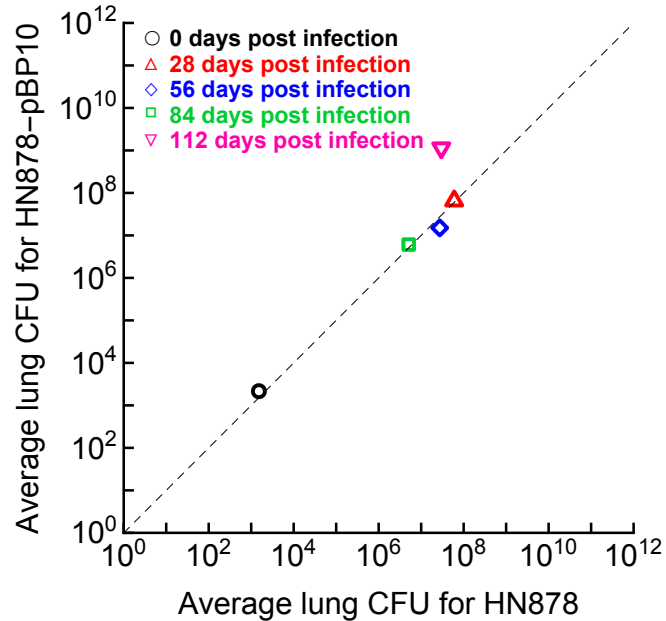**B**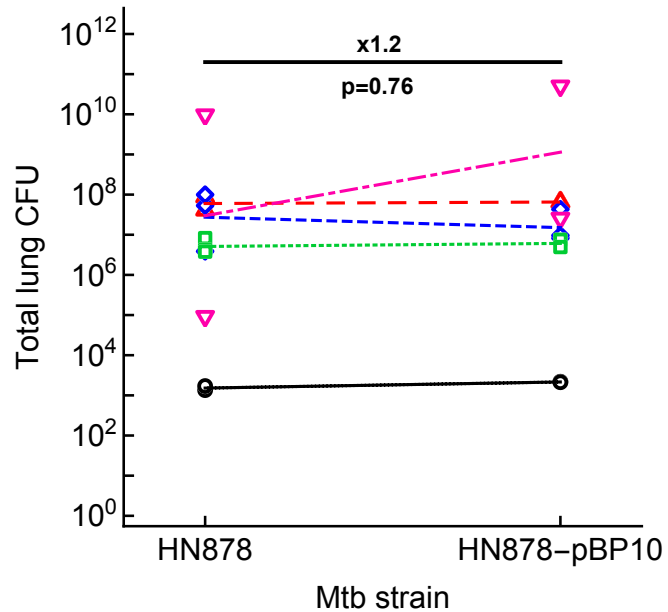

Supplement: S3 Fig — We infected rabbits with HN878 or HN878-pBP10 and measured bacteria numbers in the whole lung at different days after infection. We plot average total number of bacteria in lungs for all rabbits (panel A) or compare lung CFU for individual rabbits (panel B). (PDF) [file pcbi.1012563.s003.pdf]

**A**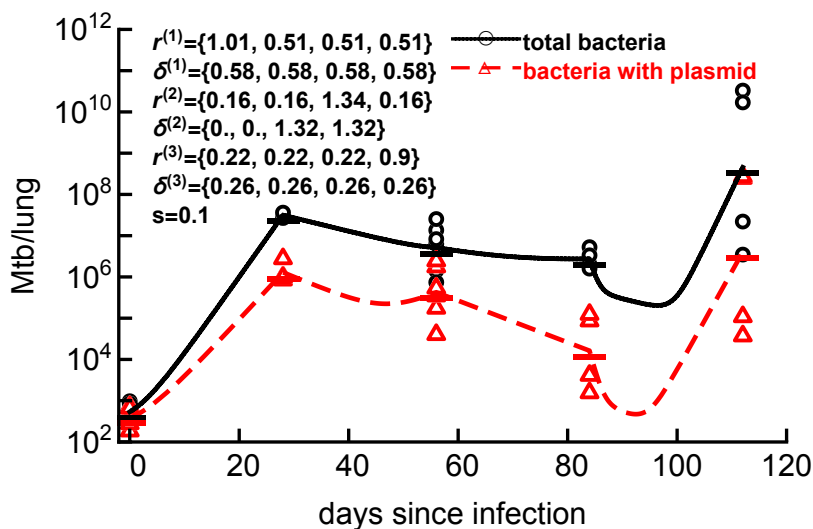**B**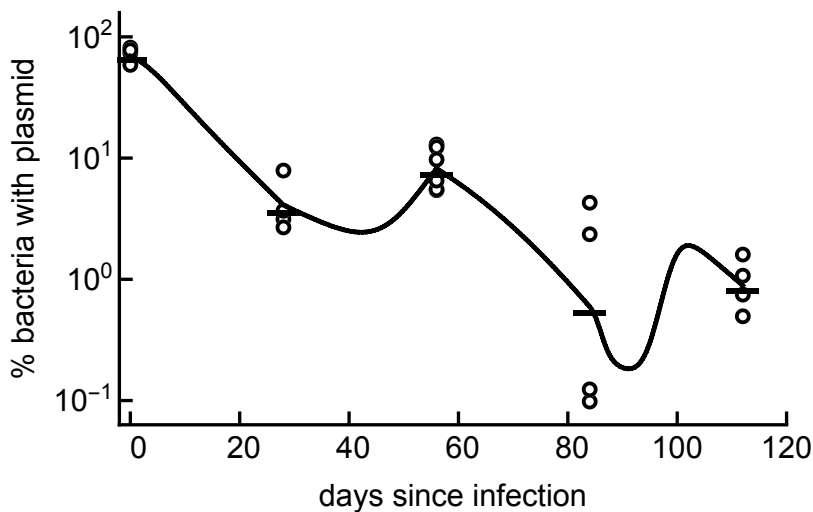**C**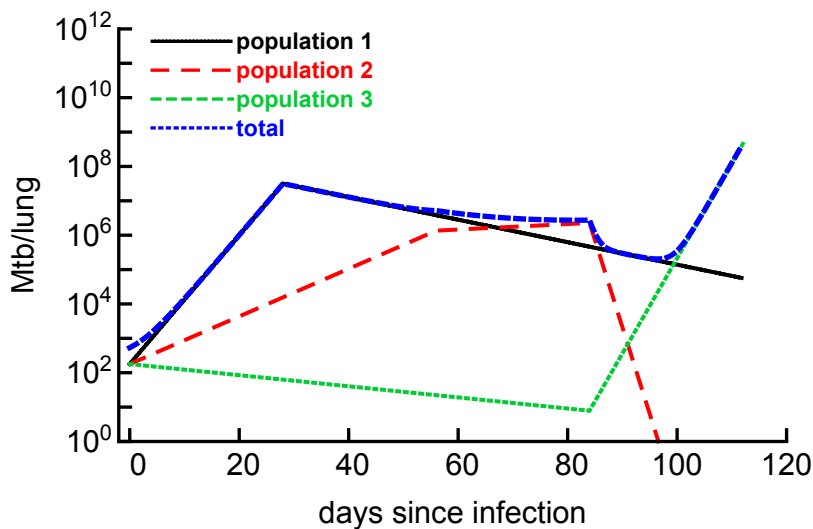

Supplement: S4 Fig — We extended the basic, one population model to include three asynchronous sub-populations of bacteria and fitted the model to the data as described in Fig 4. Label notations are the same as in Fig 4. Estimated parameters are show in panel A, additional parameters are P(1)(0) = P(2)(0) = P(3)(0) = 131.5, F(1)(0) = F(2)(0) = F(3)(0) = 50.3, σN = 0.17, σf = 0.073, L=-1633.83, AIC = −3229.53. (PDF) [file pcbi.1012563.s004.pdf]

**A**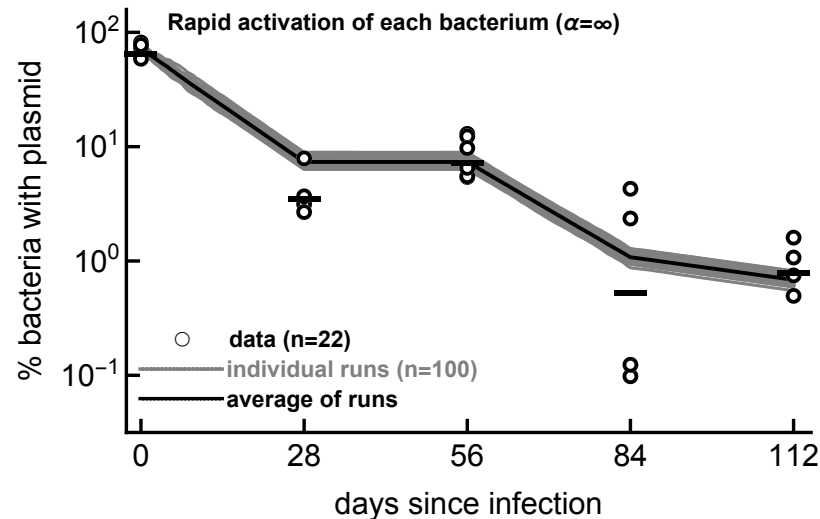**B**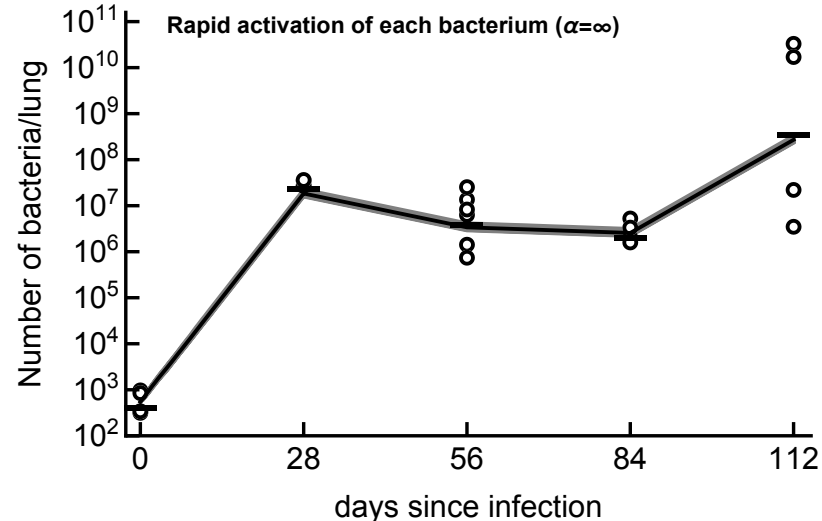**C**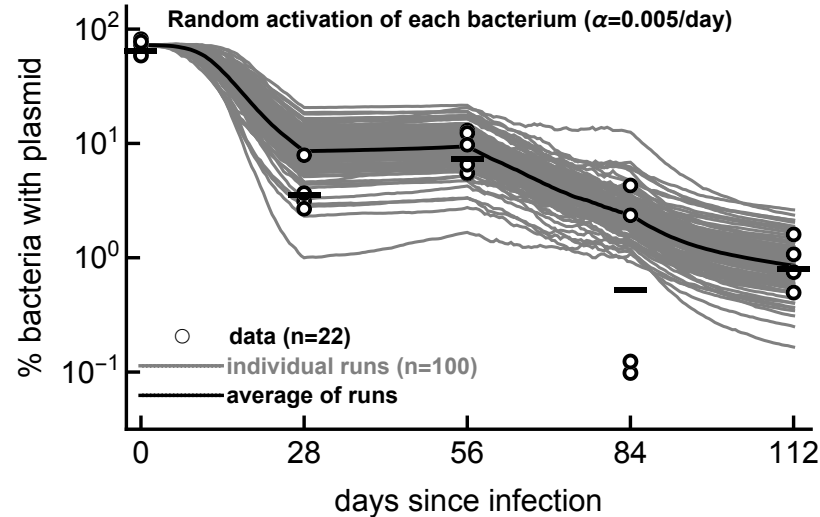**D**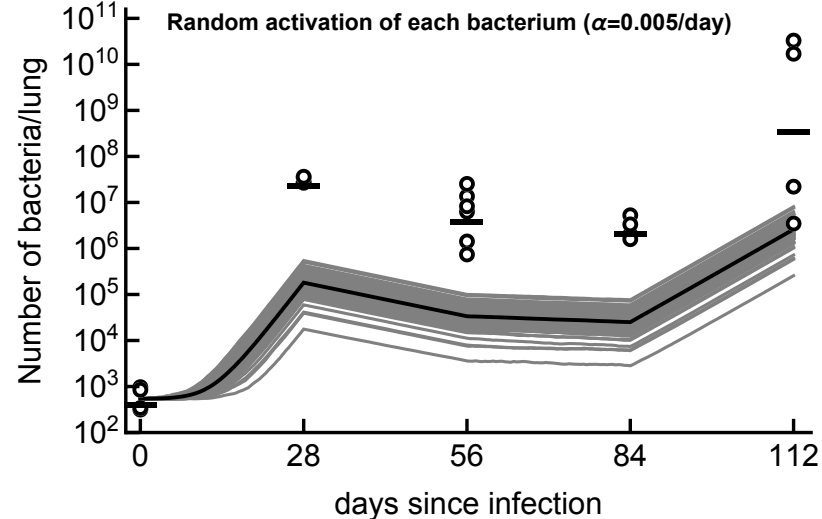

Supplement: S5 Fig — We simulated Mtb dynamics starting with 545 bacteria out of which 394 had the plasmid (as was observed in our experimental data) using parameters estimated by fitting one population model to the data (Fig 4A). In simulations we assume that bacteria start replicating and dying at day 0 (panel A&B) or that infection starts with bacteria in resting state and these bacteria become activated over time at a rate α = 0.005/day (C&D). We show the data (markers) and model predictions for the dynamics of 545 bacteria (gray lines for individual simulations and solid black lines are the averages) for the percent of plasmid-bearing cells (A&C) or the total number of bacteria in the lung (B&D). Simulations for 545 independent bacteria were repeated n = 100 times. (PDF) [file pcbi.1012563.s005.pdf]

**A**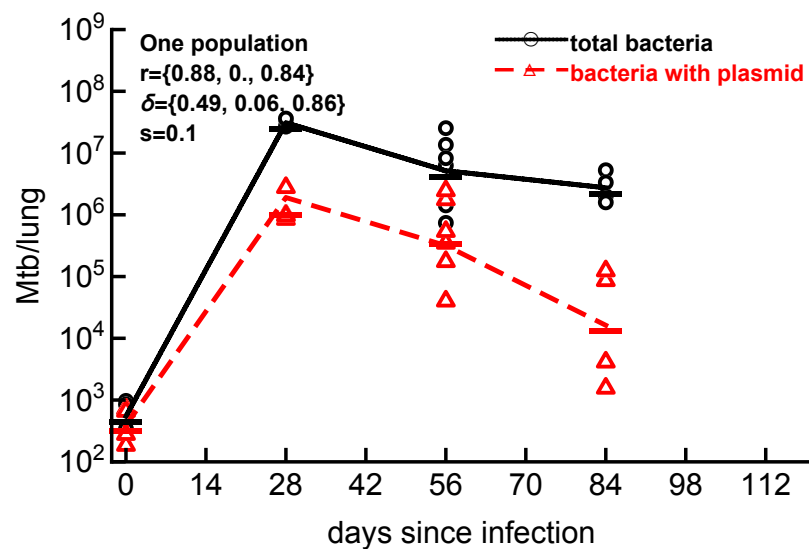**B**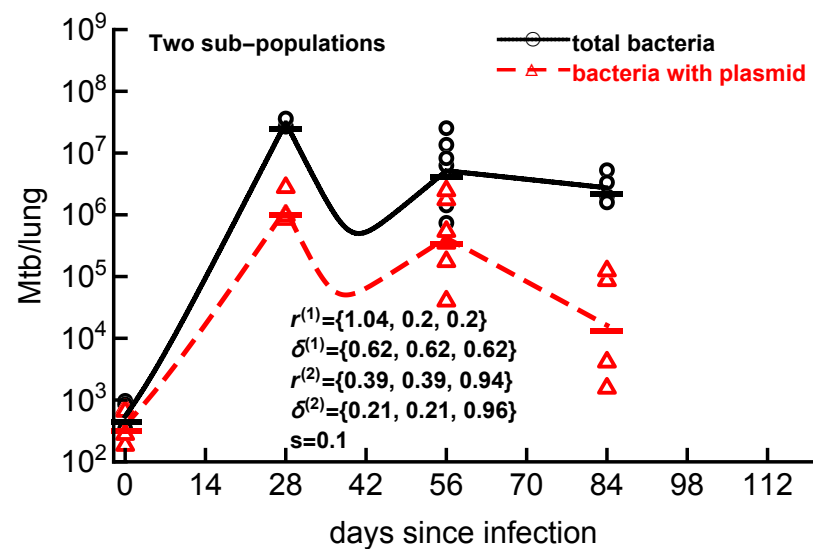**C**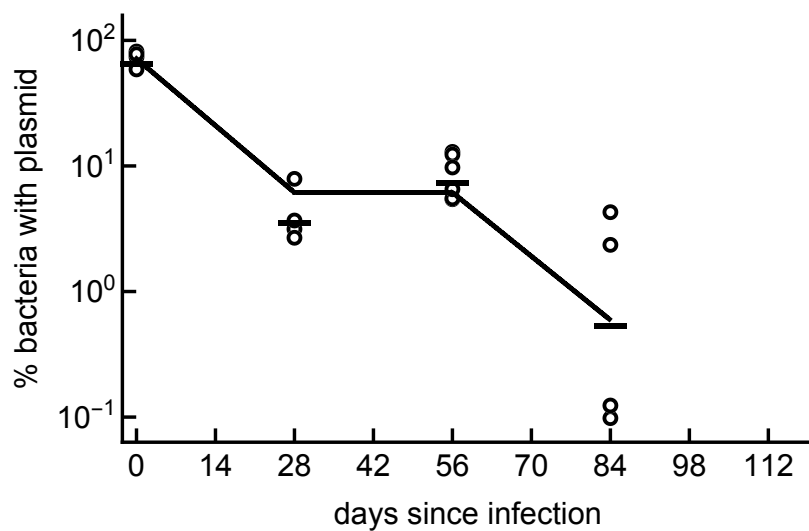**D**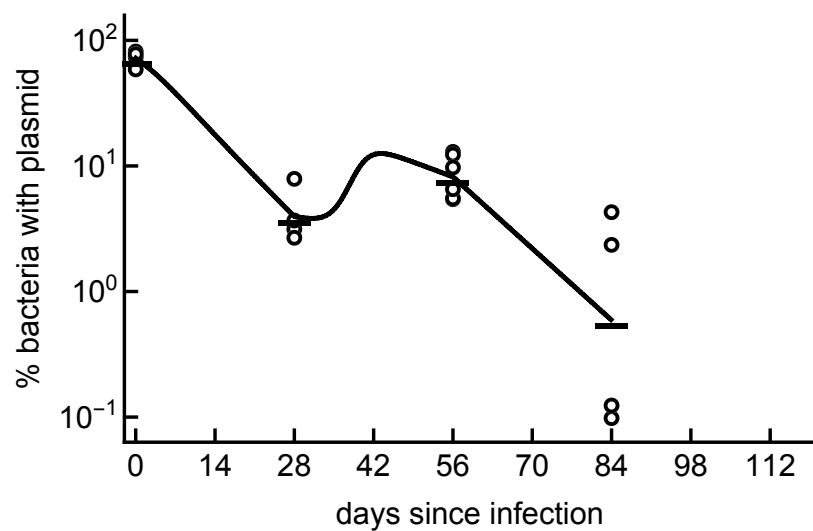**E**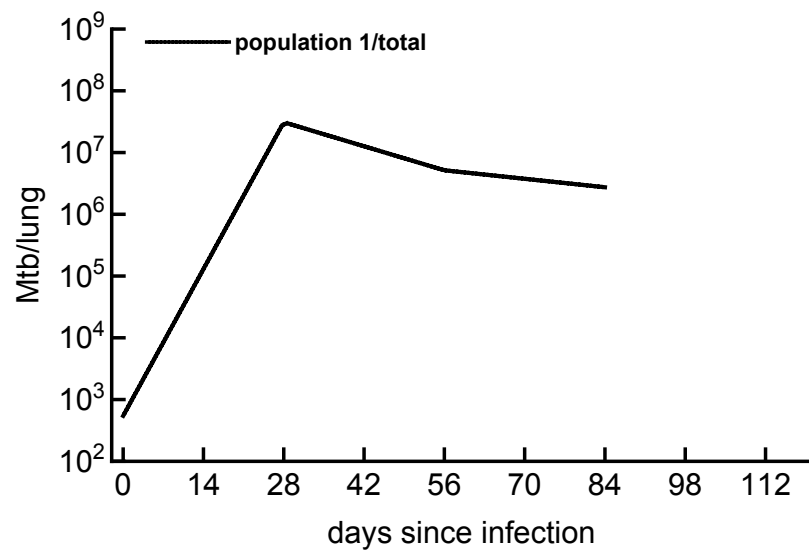**F**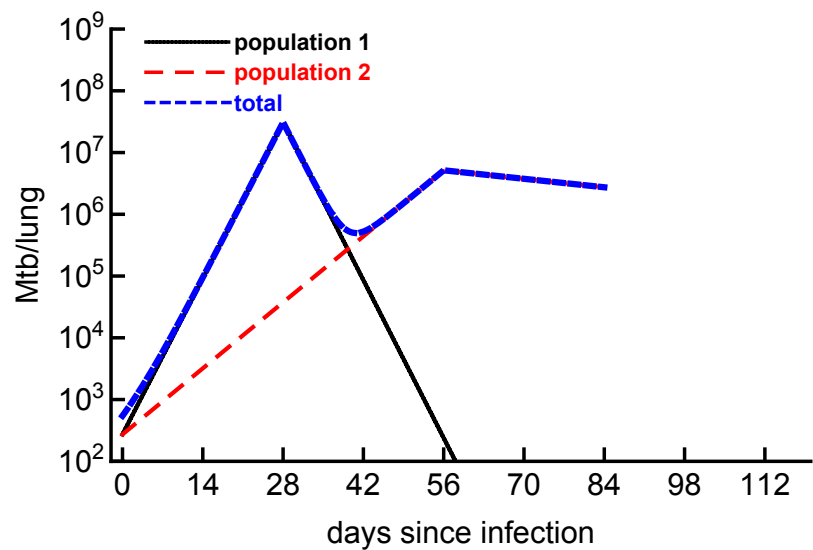

Supplement: S6 Fig — We fitted one population (panels A, C, E) or two sub-population (panels B, D, F) models to the data on Mtb dynamics in rabbit lungs for the first 84 days since infection using a likelihood approach (see Fig 4). Estimated model parameters for one population model are shown in panel A; other parameters are P(0) = 393.4, F(0) = 151.8, σN = 0.081, σf = 0.091. The negative log-likelihood of the fit was L=-1267.65. While fits in panel A look reasonable, the model is unable to accurately describe increase in the percent of plasmid-bearing cells between 28 and 56 days since infection (panel B). Estimated model parameters for two sub-populations model are shown in panel B; other parameters are P(1)(0) = P(2)(0) = 197.2, F(1)(0) = F(2)(0) = 75.5, σN = 0.081, σf = 0.087, L=-1282.78. The fit with two sub-populations is significantly improved as compared to the fit with one population model (χ12=30.3, p ≪ 0.01). (PDF) [file pcbi.1012563.s006.pdf]

**A**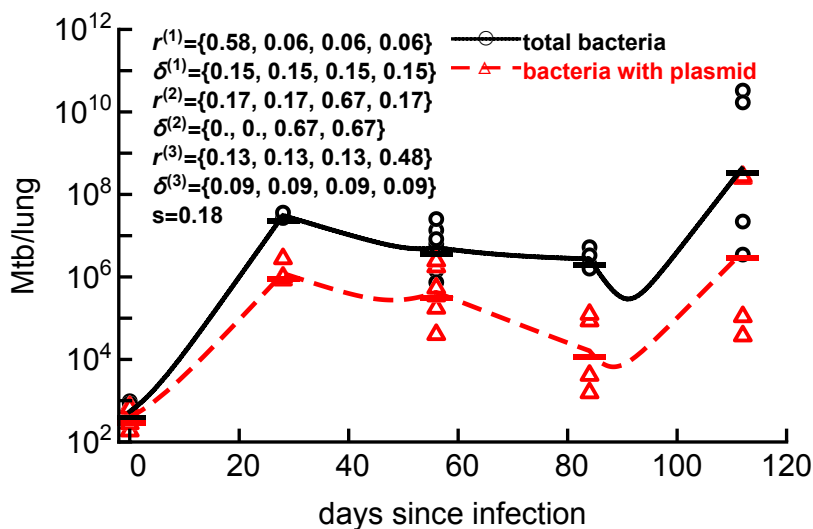**B**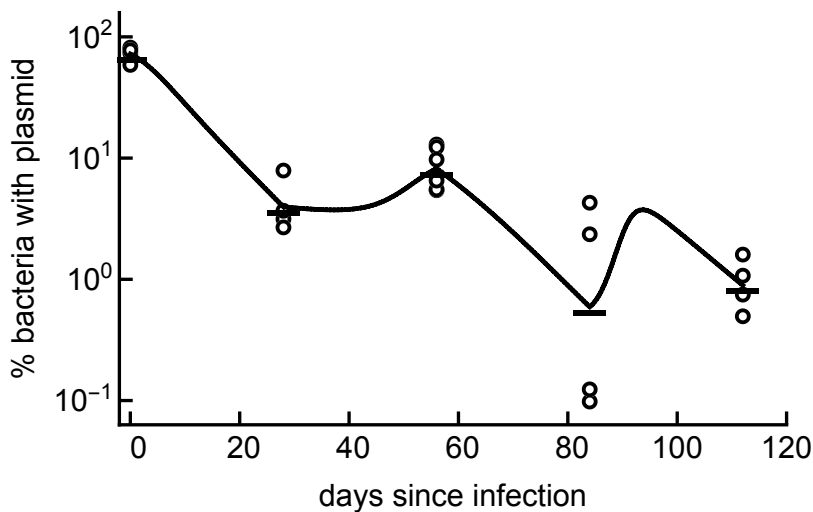**C**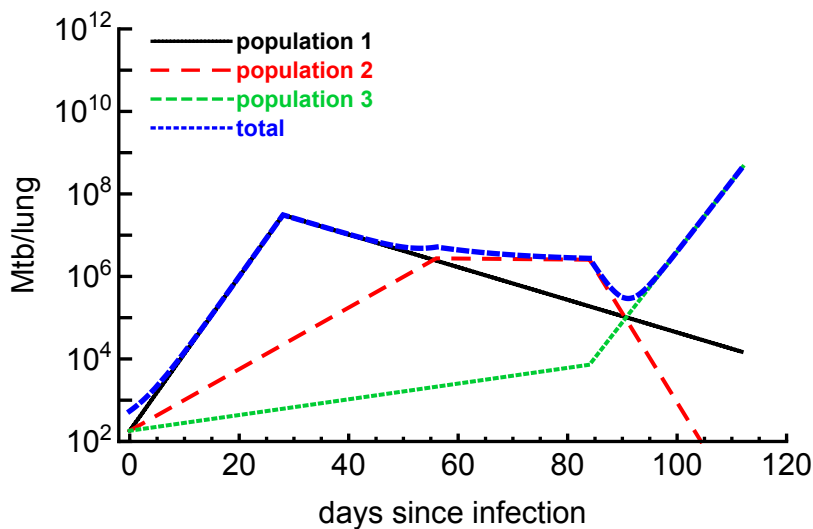

Supplement: S7 Fig — We extended the basic, one population model to include three asynchronous sub-populations of bacteria and fitted the model to the data as described in Fig 4 assuming that s = 0.18. Label notations are the same as in Fig 4. Estimated parameters are show in panel A, additional parameters are P(1)(0) = P(2)(0) = P(3)(0) = 131.5, F(1)(0) = F(2)(0) = F(3)(0) = 50.3, σN = 0.17, σf = 0.073, L=-1634.04. (PDF) [file pcbi.1012563.s007.pdf]

**A**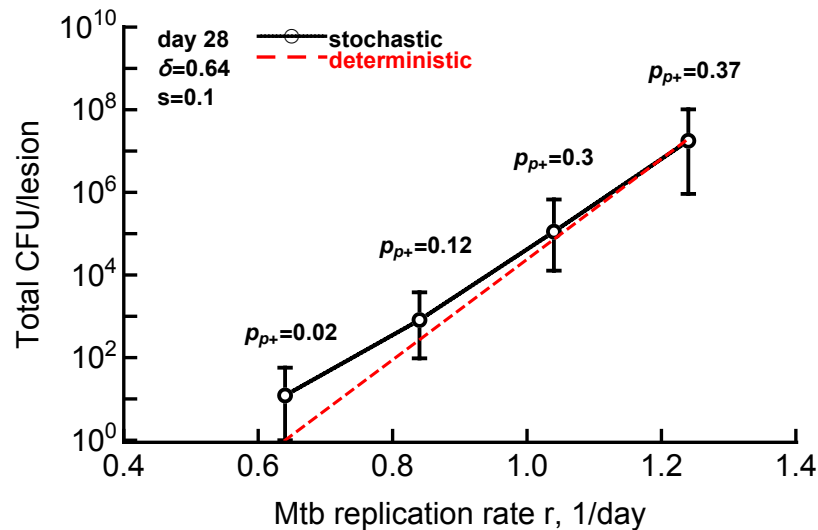**B**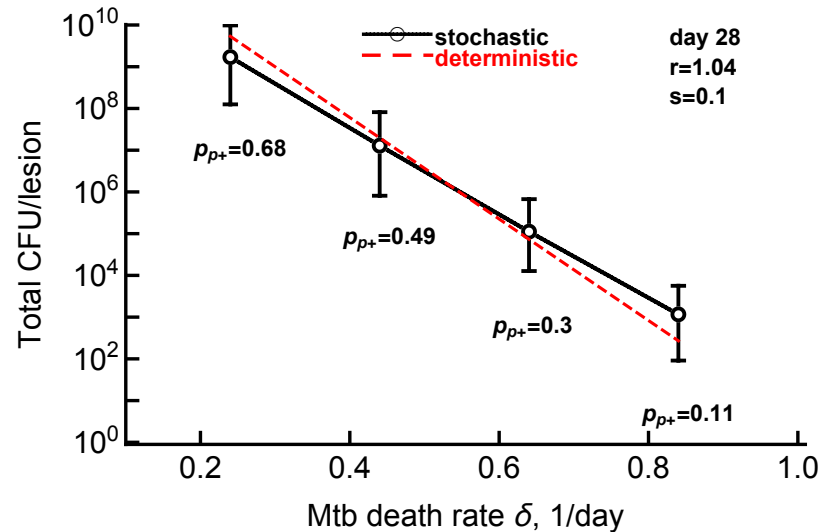**C**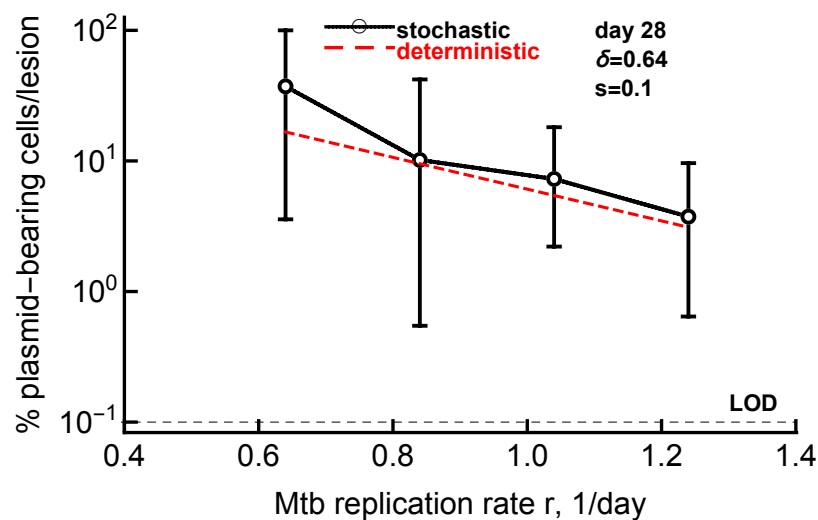**D**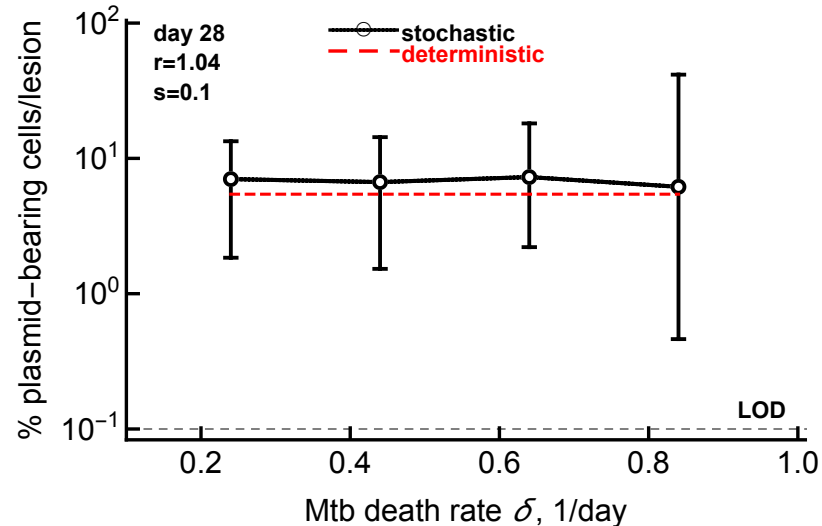

Supplement: S8 Fig — We performed simulations similarly as in Fig 5 but by varying replication rate r or death rate δ independently. We selected trajectories in which the percent of plasmid-bearing cells was above LOD. We show how the total number of bacteria (panels A-B) or the percent of plasmid-bearing cells (C-D) per lesion changes with increasing replication rate (A&C) or death rate (B&D). Bacterial numbers are sampled at 28 days (4 weeks) post-infection. (PDF) [file pcbi.1012563.s008.pdf]
