# Supplementary material for: Mathematical modeling suggests heterogeneous replication of Mycobacterium tuberculosis in rabbits
Source: PLoS Comput Biol. 2024 Nov 25;20(11):e1012563. doi: 10.1371/journal.pcbi.1012563 (PMC11627432; doi:10.1371/journal.pcbi.1012563)
Supplement: Attachment — Submitted filename: response-PLoS-CB-ver4.docx [file pcbi.1012563.s009.docx]

**Response to the reviewers’ comments**

In our response letter, we first cite the comment, give our response and provide (when necessary) updated text of the revised manuscript.

**Reviewer 1.**

*In this paper the authors report results of experiments of Mtb infection of rabbits and modeling of the within rabbit dynamics of the number of bacteria, especially replication and death rates*.

**Response**. Thank you for succinct summary.

*Line 71 (and other places): “enters in static equilibrium” or “enters a static equilibrium”?*

**Response**. Thank you, changed.

*Line 78: “at such high dose” is perhaps better than “at such higher dose”.*

**Response**. Thank you, changed.

# *Line 149: It is a little confusing that you say slope of N and P can be used to calculate division and death rates using equations (1) and (2), when these equations don’t include slope of P. It would also be useful to briefly re-state that the form of these equations was derived/presented in Gill et al [17].*

**Response**. Indeed, confusing. What we meant is that we only can use slopeN and slopeP (instead of slopeNi and slopeFi) to estimate r and delta. We rewrote it to the following by adding reference to the pioneering study of Gill et al:

“If s is known by using slopes slopeNi and slopeFi it is straightforward to estimate the rates of Mtb replication and death from eqns. (1)–(2) as was originally proposed in Gill et al. [17]. The rates of Mtb replication (r) and death (δ) can be also estimated using SlopeNi and SlopePi (the rate of change of log of the number of plasmid-bearing bacteria) [22].”

# *Line 153: You say that Mtb is expected to not die in vitro, do you also check for this in the experiments, e.g. Mtb viability? Why do you use the two media, what is the difference?*

**Response**. We followed the ideas proposed by Gill et al. 2009 where they used in vitro culture to estimate the segregation coefficient *s*. We did not look at cell viability in those experiments but in our experience, in reasonably good conditions Mtb do not seem to die in vitro within the experimental time points as we mentioned in the text. The use of two media was done to force Mtb to replicate at different rates, again, following a design of Gill et al. study. We added the following sentence in the Materials and methods:

“Specifically, Mtb HN878-pBP10 was grown in vitro starting with 2 × 10^7^ cell/mL in 10 mL for 3 days in 2 different media: complete Middlebrook 7H9 media (“7H9”), 1:3 7H9:PBS diluted media (“1:3 7H9”) to simulate different replication rates of the bacteria as previously described by Gill et.al.”

# *Line 197: “use the method”*

**Response**. Thank you, done.

# *Line 204: This seems to indicate that you expect the fit to be more stable if you use N and f, rather than P and F. Why is that?*

**Response**. We do not have a solid statistical answer. What we can say is that in our preliminary analyses we found that because of CFU variability the difference in fits by 1 population model vs. 2 populations model were not impressively different when just looking at P and F (or N and F), e.g., when measured by the sum of squared residuals (**SSR**). However, differences were quite large when we used the percent f and the total number of cells. Intuitively, the percent f is a more sensitive metric and is directly related to the replication rate, so this is why we opted for fitting the model predictions for N and f to data.

# *Line 305: It is unclear what you mean by negative rates here. Negative r and negative delta? Please explain what that means… This was explained just below, perhaps a call to “see below” would be enough here.*

**Response**. Added “see below” as suggested.

# *Line 358: Perhaps it is more accurate to say that “Mtb population consists of at lest two sub-populations of bacteria.”, since it is not clear if the model suggests two populations for biological of statistical reasons.*

**Response**. Added “at least”. And of course, it is a statistical argument here. Biologically, presence of multiple sub-populations is easy to envision. Just by scRNAseq analysis on individual bacteria, one can find tens of “sub-populations” as many studies have done with immune cells.

# *Line 400: You provide the AIC, but not the qui-squared or p-value for the comparison of two vs. three populations.*

**Response**. We cannot use likelihood ratio test here because these models are not nested anymore –as mentioned in the text, we try to constrain different sets of replication/death rates for different time periods. Therefore, 2 or 3 sub-population models are always nested with 1 population model but not between each other. (They are in general case but not in specific cases with constrained r and delta.)

# *Line 465: “are likely to be formed by”, the word “be” is missing.*

**Response**. Thank you, done.

# *Line 487: This line reads awkwardly, is the word “are” extra?*

**Response**. Yes, “are” was removed.

# *Line 530: Should this be “about 100x more lung weight” and not 1000x?*

**Response**. Indeed, thank you. The CFU comparison, however, is correct (used 100x factor here).

# *The paper is well written overall, and the methods are explicit. Please consider my suggestions above.*

**Response**. Thank you for careful reading of the paper and feedback on the text. We really appreciate it! (Several people read the paper prior to submission and yet missed those, so again, thank you!)

# *In addition, a couple of other points: i) what is your estimate of total bacterial replication and death, in absolute numbers, over the ~4 months of the experiment? How many bacteria were produced and killed over this period?*

**Response**. This is an interesting question. We approached it by simulating Mtb dynamics in accord to our best fit 2 sub-population model assuming no death rate (delta=0). We find that by the end of 16 weeks of experiment, we should expect to see 3.9*10^36 bacteria, but we only see 4.5*10^8 CFU/lung. So, vast majority of bacteria that could be produced in a rabbit are eliminated, most likely, by the host immune response.

# *… ii) a second question, is whether heterogeneity in s (either across bacterial population or over time) could also explain your data?*

**Response**. Yes, it can if s varies with different sub-populations. Because replication rate and s enter the equation for plasmid loss as a product, variability in either could be an explanation. We have explored the role of different s values on the estimates of the replication and death rates in our previous work (McDaniel et al. Front Microbiol 2016). We have added the following point in our Discussion:

# “While we propose that to explain the data, Mtb population must consist of several (at least 2) semi-independent sub-populations, we do not know what these sub-populations may represent. One possibility is that these sub-populations may reside in different areas of the lung with variable access to oxygen, other nutrients, or different access by the immune response-mediated bacterial clearance [46]. It also could arise from different subpopulations within individual granulomas, e.g., cells may have different replication kinetics when they are intracellular vs. extracellular. Heterogeneity may arise due to segregation coefficient s being variable in different Mtb sub-populations. Using Mtb strains with the replication clock plasmid, sorting different cell sub-populations and using other methods to evaluate Mtb replication rate (e.g., [47]) may help discriminate between these alternatives.”

# *iii) what if s was not independent of replication rate, is this a possibility?*

**Response**. Gill et al. experiments clearly showed that s was independent of the rate of Mtb replication in vitro. It was also true in our experiments (Figure 2E). Now, it is possible that s is dependent on the rate of Mtb replication in vivo. Then of course, our conclusions would probably need revision but for that we need to know how s is dependent on the Mtb replication rate. As mentioned above, we explored this possibility in our previous study (McDaniel et al. Front Microbiol 2016). The way forward here is to use other ways to estimate rates of Mtb replication and death in vivo, e.g., by using measurements of CFUs and chromosome equivalents (CEQs) or RS ratio (Munoz-Elias et al. IAI 2006; Lin et al. Nat Med 2014; Walter et al. Nat Commun 2021). We are currently developing mathematical models for the latter metrics; comparing estimates of the Mtb replication and death rates using replication clock vs. CFU/CEQ is something we would like to do with new funding. The following is what we now added in the Discussion:

# “The key assumption in our analysis is that the segregation coefficient s is independent of the rate of Mtb replication, so that the percent of plasmid-bearing cells declines at a rate proportional to the replication rate (eqn. (6)). While the evidence that s = const in vitro is relatively strong (Figure 2E and [17]), it is still unknown whether s varies with time or between different Mtb sub-populations in vivo, particularly in heterogenous granulomas such as those found in the rabbit model of TB. We have previously shown that change in segregation coefficient s with time dramatically impacts estimates of the rates of Mtb replication and death [22]. Future studies will need to use different methods (e.g., based on integration of replication clock plasmid with Mtb RS ratio or CEQ measurements, [17, 18, 23, 30]) and evaluate whether these different metrics give similar estimates of Mtb replication rates and death in vivo.”

# **Reviewer #2:**

# *Key question: whether variability in granuloma-scale control of bacteria arises due to differences in replication rates or death rate (or both) of bacteria in different lesions remains largely unknown. They suggest it is the balance of death and division rates—and base it nothing on the role of immunity holding things in check. This is surprising and very simplified version of what could be happening.*

**Response**. Based on this and several other comments, it seems to us that the reviewer misunderstood our definition of parameters r and delta in our model (and model of Gill et al.) mean. These are not “just replication” and “just death” rates, these are parameters that cumulatively describe impact of various factors, including of course immune response, on ability of Mtb to persist (r = delta), grow (r>delta), or be eliminated (r<delta) in the host. We could not explicitly model immune response simply because we do not have measurements of immunity in these experiments with HN878-pBP10 in rabbits. Our plan is to apply for funding and measure Mtb-specific immune responses in individual granulomas, and then extend the model to specifically include immune response dynamics as we measure it. Including hypothetical immune response dynamics without having solid experimental data is not a good way to build models as this will result in overfitting and may be misleading.

*The premise of the is paper is that “It is well understood that pulmonary TB is due to replication of Mtb in the lung but quantitative details of Mtb replication and death in lungs of patients and how these rates are related to the degree of lung pathology are unknown” and this point is certainly controversial. Many experts believe that the immune response to Mtb bacteria within lungs is what lead to TB disease manifestations so the premise that replication as the sole factor is misinforming.*

**Response**. Again, the reviewer seems misinterpreting what we mean by Mtb replication in our model. First, if one bacterium lands in a human lung cell and does not replicate, it will eventually die and there will be no tuberculosis (TB). So, replication is clearly required. Now, if bacteria replicate, will TB ensues with 100% certainty ? We don’t know; but if bacteria reach some numbers, there will be immune response, although the outcome of such immune response being unclear. Infection can be cleared (typically this is not well understood), could persist (latency), or could cause active TB within short period of time (6-12 months). But it is clear, that Mtb replication is required for that, i.e., bacteria must divide. However, it is still unclear if the rate of Mtb replication (that is also influenced by immune response) relates to TB severity; hence, we need methods to estimate how quickly bacteria replicate and die in vivo. We do believe that the immune response to Mtb bacteria within lungs does impact the bacterial replication and death, which manifests in the differential clinical outcome.

# *Other concerns/questions--*

# *Regarding the different kinetic groups of bacteria, it is important to note that bacteria reside in 3 major areas within lung granulomas: 1) trapped with macrophages, extracellular and those that are non-replicating within the caseous necrosis that are still metabolically active but not dividing. There is no discussion of this.*

**Response**. Our data are for total lung CFU (i.e., divided into right and left lungs), so these elements are not relevant. As the reviewer commented, the exact location of Mtb within the lung is important to understand the disease biology but because in our experiments we have not measured these intricate and difficult-to-measure sub-compartments, discussing these details will derail the readers from key points of our study. We did include this element in the Discussion as limitations of our work as follows:

“The models we have developed and fitted to data lack many biological details about Mtb dynamics in vivo. For example, it is well understood that in granulomas, Mtb residing in different regions (i.e., inside of macrophages, in caseum, etc) may have different replication capacities [46]. While we estimated the rates of Mtb replication and death we did not investigate how the immune response in various in vivo compartments controls these rates. In part, this is because measurements of Mtb-specific immune responses were not done in our experiments. To gain better insights into how immunity controls Mtb dynamics, our future work will focus on correlating estimated replication and death rates with magnitude of immune response components in individual lung granulomas of rabbits.”

# *Where and why do these subsets exist?*

**Response**. Because our data do not include these subsets, explaining where and why the subsets exist would be hypothetical and is beyond the scope of this paper.

# *Continual references to the mouse model are made and all know that the mice is NOT a good model for TB and in fact, they get chronic infection which does not happen in primates or rabbits. There is also a paper on the ultra-low dose mouse model (urdalh • PMCID: PMC7854984 ) that shows that it is closer to what happens in primates than the 100 dose model that the authors refer to.*

**Response**. We referred to the original article by Gill et.al that used a B6 mouse model to validate Mtb dynamics using the “replication clock” plasmid-containing bacteria. Since we also used the same system to generate CFU data from rabbits to model, we cited that original study. We do acknowledge that each animal model is different in their contribution to our understanding of TB pathogenesis; however, we are not comparing the Mtb dynamics in different species in our current article

# *Line 48,52 chronic stage of infection. —this seems to be only present in mouse models. Reference [26] points out the three relevant animal models for TB*

**Response**. We are unsure of the definition used by the reviewer for “Chronic infection”. However, we have seen disease pathology and bacterial presence for prolonged period of time (i.e, upto 16 weeks post infection) in our rabbit model of active disease – please see Fig 3B in our paper and PMID:23958185, 22960076.

# *Line 60 isoniazid treatment works but the author did not explain that it takes MONTHS for that to happen…and that gives the bacteria opportunity to change replication rates in that time frame.*

**Response**. We acknowledge that isoniazid treatment works to eradicate actively replicating bacteria. However, the point we want to emphasize in this article is that if bacteria were NOT replicating during this stage of infection, isoniazid would have no impact. Clinical trials clearly showed that isoniazid prevents progression to active TB, so bacteria must be replicating during that 6-9month treatment period and thus are not dormant (non-replicating). However, there is no experimental evidence in the literature to show that the bacteria are synchronously changing its replication rate in the time frame between the start of INH treatment and months-after treatment.

# *Almost all TB scientists believe that Mtb is replicating during LTBI and that it is the immune response that keeps it in check.*

**Response**. We are uncertain about the replication dynamics of Mtb during LTBI. But we believe that the outcome of Mtb infection ranges from full clearance, dormancy, active persistence/replication, and symptomatic disease. This is in-line with the recent agreement in the field to go away from the term “LTBI”, and towards a broader term, “spectrum of outcomes” (e.g.PMID: 38527485).

# *Perhaps the reason that the previous model failed to represent the data is that they are ignoring the large piece of immune control? And as stated it is well-accepted that there are multiple subpopulations of Mtb in a host (see any paper on PK/PD of treating TB)*

**Response**. As we explained above, immunity has been incorporated indirectly in our mathematical models as immunity is expected to influence Mtb parameters r and delta. It is true that heterogeneity of Mtb population is cited to explain why 4 drugs are typically needed to cure people with TB; however, such multiple subpopulation of Mtb are not experimentally reported in the rabbit model with active disease, even though it is perhaps expected. Now we show that to explain our data in the rabbit model, some level of heterogeneity in Mtb replication and/or death is needed. This perhaps further confirms the relevance of rabbits as a more faithful model to study human TB.

# *Their authors have not made it clear that while their mathematical model can track different replication rates, it does not explain that OTHER FACTORS are at work influencing the dynamics. For example, these authors are well-acquainted with the relational to a viral dynamics of HIV/HepC and the viral set point has a very similar relationship to the MTb setpoint in TB. (in primates and rabbits, not mice). Thus, the leveling off of bacteria is driven by the immune response, as is the viral replication load in these other infections. How can those features not be included even phenomenologically in their new model?*

**Response**. We believe that the immune response to Mtb is a highly complex, multifactorial phenomena, and including dynamics of immune response for the bacterial CFU data is premature because we have no data on the type and kinetics of immune response dynamics in these rabbits. As the reviewer pointed out, those parameters will be evaluated in our future studies.

# *Line 153--Is the assumption of “In vitro we expect that bacteria do not die (i.e., delta = 0) Assumption for all time or only the length of the experiment? How useful is this assumption when an immune response is present?*

**Response**. This is expected for some time of in vitro experiments, as it was also assumed in Gill et al. Nat Med 2009 paper. Since there is no immunity in in vitro culture, this assumption seems to be reasonable for at least several weeks. Obviously, the in vivo immune response impacts bacterial replication and death disproportionately, which is irrelevant to our assumptions made in vitro for the modeling.

# *Line 189-“ We propose that a non-monotonous change in the percent of plasmid-bearing cells in the bacterial population may arise due to asynchronous dynamics of several independent su populations of bacteria.” -What would lead to these different independent populations? Why would they exist?*

**Response**. As the reviewer might agree, the generation and maintenance of different Mtb population and their role on pathogenesis were not fully understood. We can only hypothesize; one idea is that these sub-populations may be located in different areas of the lung (e.g., upper vs. lower lobes, specific area in the lobe, etc) that may have different access to oxygen, blood, etc. Gravity could be an important factor determining how easily fluids (blood, lymph) have access to specific sites in the lung (6839825). To clarify this, we wrote the following in the Discussion section:

“While we propose that to explain the data, Mtb population must consist of several (at least 2) semi-independent sub-populations, we do not know what these sub-populations may represent. One possibility is that these sub-populations may reside in different areas of the lung with different access to oxygen, other nutrients, or different access by the immune response. It also could arise from different subpopulations within individual granulomas, e.g., Mtb may have different replication kinetics when they are intracellular vs. extracellular. Using reporter Mtb strains with the replication clock plasmid and sorting different cell sub-populations may help discriminate between these alternatives.”

# *not know why the dynamics of bacterial growth changed after 6 days of culture; it may be related to the adaptation of the natural Mtb isolate HN878 to the growth in culture. This dynamics is not consistent with the continuous monotonic increase in the concentration of H37Rv-pBP10 in 7H9 media of different dilutions (e.g., Figure 1d in Gill et al. [17]). -This is very strange to hang the whole story on this in vitro culture and no real understanding is given for the dynamics?*

**Response**. The experiments to generate real-world data to model the dynamics of Mtb growth are not easy, and we have repeated them few times, we always saw some variability in bacterial dynamics, including our more recent experiments with another Mtb strain CDC1551-pBP10. This is in sharp contrast with the Gill et al. study that generated much prettier curves. In the future, we plan to understand this point further but at present we cannot offer a solid explanation without additional experimental data that will require additional funding to generate.

# *Line 290- “500 to nearly 108 in the  first 28 days, then the number of bacteria declined for the next 56 days (until 84 days), and then the number of bacteria exploded to about 1010 CFU/lungs in some rabbits or was maintained at 107 CFU/lungs in others..*

# *-this is exactly tied to the “bacterial set point idea” raised above and the timinig is the length of time for the generation of adaptive immunity in rabbits to indice the turn around at day 28. The explosion in bacteria is due to Rabbits being a progressive model of TB and the immune response is unable to control infection.*

**Response**. We believe that this “set-point” idea is interesting but because our models do not explicitly include the dynamics of Mtb-specific immune response (due to lack of data), we do not feel comfortable addressing this point. But we would like to thank the reviewer for the idea, and we will explore it in our future studies.

# *-the authors could not explain the results with the original model of Gill et al so build another two-population model (although it is likely 3 as mentioned above) and show it could work. There is no basis for this 2-sub pop model and again, could a model of immune response and Mtb (similar to the HIV models) explain the same thing?*

**Response**. Well, just above this reviewer strongly argued that having Mtb sub-populations is obvious in drug PK/PD studies, and as we suggested above, physical location of different lesions could be one reason for different “sub-populations” – but as we state in discussion, this hypothesis will have to be tested with experimental data in the future. Including immune response in 1 population model will not work because ultimately, immune response will influence r and delta, and it will not be possible to explain increase in percent of plasmid-bearing cells with such a model. We made all our data available (on github), the reviewer is welcome to try generating a model with immune response dynamics and 1 population that would explain the data (Fig 3B-C) with good quality. However, the focus of our current manuscript is to rigorously evaluate bacterial dynamics in Mtb-infected rabbit lungs, for the first time in literature. We will elaborate and extend the findings of this study to include different parameters as the reviewer has suggested,

# *Line 330-And predicted no Mtb replication between 28 and 56 days post infection. -which of course there is no evidence for, and it is the immune system that is keeping it in check.*

**Response**. We disagree with this comment. Actually, the one population model predicts r=0 and delta >0 (decline in CFU), not r=delta (which would imply control). Indeed, CFUs are actually declining during this time period (Fig 4A).

# *Line 377” Then to explain the increase in the percent of plasmid-bearing cells, while the total number of bacteria declines between 28 and 56 days post infection, one must assume that the initial dominant population contracts which another population which another population with a higher frequency of plasmid-bearing cells (due to fewer divisions) becomes more dominant*

# *—again what is the mechanism or driving biology for this segregation? Perhaps their results of the two-pop sub model could also be explained that some bacteria are being taken up by macrophages and some are being trapped in caseous necrosis? The environments lead to different replication rates as well as any that remain extracellular. Rather than a new assumption*

**Response**. The points that the reviewer has mentioned above are a possibility, thank you. We now list that in the Discussion as follows:

“While we propose that to explain the data, Mtb population must consist of several (at least 2) semi-independent sub-populations, we do not know what these sub-populations may represent. One possibility is that these sub-populations may reside in different areas of the lung with different access to oxygen, other nutrients, or different access by the immune response. It also could arise from different subpopulations within individual granulomas, e.g., bacteria may have different replication kinetics when they are intracellular vs. extracellular. Using reporter Mtb strains with the replication clock plasmid and sorting different cell sub-populations may help discriminate between these alternatives.”

# *Line 398 Interestingly, while the three population model  fitted the data statistically better than either a single population model*

# *-As expected as 3 different populations are well-documented…and this gives biological plausibility to their argument. Not discussed and should be.*

**Response**. Thank you, see our addition to the discussion section above.
