# Supplementary material for: Mathematical modeling suggests heterogeneous replication of Mycobacterium tuberculosis in rabbits
Source: PLoS Comput Biol. 2024 Nov 25;20(11):e1012563. doi: 10.1371/journal.pcbi.1012563 (PMC11627432; doi:10.1371/journal.pcbi.1012563)
Supplement: Attachment — Submitted filename: response-PLoS-CB-ver6.docx [file pcbi.1012563.s010.docx]

**Response to the reviewers’ comments (round 2)**

In our response letter, we first cite the comment, give our response and provide (when necessary) updated text of the revised manuscript.

**Editor**

*Reviewer 2 notes that you have replied extensively to the reviewers comments but only made small edits to the manuscript. I would suggest you can and should do a better job editing the manuscript to address some of the issues rather than only replying to the reviewers. Please do so and I believe the manuscript will be acceptable for publication.*

**Response.** We went back to our responses with the previous submission and updated the text by expanding our arguments and explaining the modeling framework we used. Most of the comments were related to the interpretation of results (what do 2/3 sub-populations mean), and thus, have been primarily listed in the Discussion section of the revised paper.

**Reviewer #1:**

*Thank you for reviewing the manuscript and carefully answering my previous questions. I think the manuscript has improved and I don't have further questions.*

**Response**. Thank you.

*… In your response to one of my previous questions, regarding estimating total bacteria replication and death, you calculated this by making delta=0. I am not sure this is the best way. I would use the model simulation with the estimated delta and simply calculate the integral of production and death (for instance with two new accounting compartments for rP and deltaP, etc). This is because without death, rP is going to be larger at every time, which generates higher number of bacteria in a compound effect. I realize that you decided to not add this calculation to your manuscript, so this is a side note.*

**Response**. We would like to thank the reviewer for this note. As suggested by the reviewer, we re-calculated the total number of bacteria produced and died in our two-subpopulation model as $\int_{0}^{112} r_{i}\left( t \right)N_{i}$ and $\int_{0}^{112} \delta_{i}\left( t \right)N_{i}$, respectively. The total number of bacteria produced in 1^st^ and 2^nd^ subpopulations are 3.3*10^8^ and 4.8*10^8^, respectively (total 8.1*10^8^), and total died bacteria are 3.3*10^8^ and 3.4*10^7^, respectively (total 3.6*10^8^). These calculations now highlight that the second subpopulation contributes to most of the bacteria produced during the infection and is associated with less death, explaining how this sub-population drives disease in the animals (due to a large increase in CFU in some animals, Fig 4A&B). Interestingly, Gill et al. also calculated the total number of bacteria produced during 111 days of H37Rv-pBP10 infection of mice lungs, equaling to 1.3*10^7^ CFU/ml or, assuming a lung weight of 150mg, about 2.6*10^6^ bacteria produced in the whole lung. This analysis illustrates that HN878-pBP10 in rabbits undergoes a larger number of replications than a more commonly used lab strain H37Rv in mice further highlighting differences between these Mtb strains and host species.

We write in Discussion:

“Following an analysis performed by Gill et al. [17] we calculated he total number of bacteria produced and died in each of two sub-populations j = 1, 2 in our two-subpopulation model as … and …, respectively (see eqn. (5) and Figure 1 in Materials and methods for detail). The total number of bacteria produced in 1st and 2nd subpopulations are 3.3 × 10^8^ and 4.8 × 10^8^, respectively (total 8.1 × 10^8^), and the total bacteria that died during the experiment are 3.3 × 10^8^ and 0.3 × 10^8^, respectively (total 3.6 × 10^8^). These calculations highlight that the second subpopulation contributes most of the bacteria produced during the infection and is associated with less death, explaining how this sub-population drives disease in the animals (due to a large increase in CFU in some animals, Figure 4A&B). Interestingly, Gill et al. [17] calculated that 1.3 × 10^7^ CFU/ml of total bacteria or, assuming a lung weight of 150mg, about 2.6 × 10^6^ are bacteria produced in the whole lung of B6 mice infected with H37Rv over 111 days of infection. We conclude here that HN878-pBP10 in rabbits undergoes a larger number of replications than a more commonly used lab strain H37Rv in mice further highlighting differences between these Mtb strains and host species.”

**Reviewer #2:**

*the authors gave lengthy detailed replies to the queries raised, however only very minor edits were actually incorporated into the manuscript. This review believes that some of the same confusions observed may also be observed by other readers therefore the authors should work in a little more of the replies into the ms.*

**Response.** To write our papers we use LaTeX and then to generate the difference between two files we use latexdiff. Sometimes latexdiff generates code that does not compile requiring manual edits. It is possible that with those edits some changes were marked as not “new changes”. We went back and made sure that we addressed all the raised comments in the text; we do acknowledge though that some of our changes are shorter than those provided in the reply letter. This was done to keep the readability of the text to a high level, e.g., some points are addressed in the Discussion only.

*Also, can the authors add something to the abstract explaining the relevance and importance of their work, given that they are not considering other factors that could affect bacterial growth rates in vivo such as location, immunity etc. this reviewer is still left wondering the relevance of the work.*

**Response.** This is a fair point, and we added one extra sentence in the abstract to illustrate how our work is relevant to the field of TB pathogenesis. We also added in the Discussion that heterogeneity of Mtb replication in rabbits may also help explain the response of these animals to drug treatment because TB drugs are known to impact differently rapidly and slowly growing bacteria.

We write in the abstract:

“Taken together, our new data and mathematical modeling-based analyses illustrate differences in Mtb dynamics in mice and rabbits confirming a perhaps somewhat obvious observation that “rabbits are not mice”.”

In the discussion, we write

“Treating Mtb-infected rabbits with antibacterial drugs revealed differential response of different lung granulomas to the drugs suggesting heterogeneity in Mtb replication in rabbits [46]. By using the RS ratio metric Walter et al. [47] elegantly showed differences in Mtb replication rates in different lung areas of C3HeB/FeJ mice that form well-circumscribed caseating granulomas. Here we extend these studies by showing that even when measuring Mtb numbers in the whole lung of rabbits, along with the percent of bacterial cells carrying plasmid pPB10, we can detect and to a degree quantify heterogeneity in rates of Mtb replication and death in vivo. Our results thus may further help explain the differential response of Mtb-infected rabbits to drug treatment and may help design better treatment strategies depending on the distribution of granulomas with slow or rapidly replicating bacteria.”
